# A panel of urine-derived biomarkers to identify sepsis and distinguish it from systemic inflammatory response syndrome

**DOI:** 10.1038/s41598-021-99595-0

**Published:** 2021-10-21

**Authors:** Yao Tang, Ning Ling, Shiying Li, Juan Huang, Wenyue Zhang, An Zhang, Hong Ren, Yixuan Yang, Huaidong Hu, Xiaohao Wang

**Affiliations:** 1 Department of Infectious Diseases, The Second Affiliated Hospital, Chongqing Medical University, Chongqing, China; 2 Key Laboratory of Molecular Biology for Infectious Diseases (Ministry of Education), Institute for Viral Hepatitis, The Second Affiliated Hospital, Chongqing Medical University, Chongqing, China; 3 Intensive Care Unit, The Second Affiliated Hospital, Chongqing Medical University, Chongqing, China; 4 Department of Clinical Nutrition, The Second Affiliated Hospital, Chongqing Medical University, Chongqing, China

**Keywords:** Biochemistry, Microbiology, Biomarkers, Infectious diseases

## Abstract

Sepsis is a potentially fatal condition caused by infection. It is frequently difficult to distinguish sepsis from systemic inflammatory response syndrome (SIRS), often resulting in poor prognoses and the misuse of antibiotics. Hence, highly sensitive and specific biomarkers are needed to differentiate sepsis from SIRS. Urine samples were collected and segregated by group (a sepsis group, a SIRS group, and a healthy control group). iTRAQ was used to identify the differentially expressed proteins among the three groups. The identified proteins were measured by ELISA in urine samples. Finally, all the acquired data were analyzed in SPSS. C-reactive protein, leucine-rich alpha glycoprotein-1 and serum amyloid A (SAA) protein were differentially expressed among the three groups. The adjusted median concentrations of urinary C-reactive protein were 1337.6, 358.7, and 2.4 in the sepsis, SIRS, and healthy control groups, respectively. The urinary leucine-rich alpha glycoprotein-1 levels in these three groups were 1614.4, 644.5, and 13.6, respectively, and the levels of SAA were 6.3, 2.9, and 0.07, respectively. For all three of these measures, the sepsis group had higher levels than the SIRS group (P < 0.001), and the SIRS group had higher levels than the healthy control group. When combined, the three biomarkers had a sensitivity of 0.906 and a specificity of 0.896 in distinguishing sepsis from SIRS. Urinary C-reactive protein, urinary leucine-rich alpha glycoprotein-1 and urinary SAA have diagnostic value in cases of sepsis. This initial study suggests the possibility of improved differential diagnosis between sepsis and systemic inflammatory response syndrome; additional confirmation is necessary to corroborate the findings.

## Introduction

Sepsis was first defined in 1992^1^ as a systemic inflammatory response syndrome (SIRS) caused by confirmed infection (Sepsis 1.0). The signs and symptoms of sepsis were expanded in Sepsis 2.0^2^. Sepsis is now defined as organ dysfunction caused by a systemic inflammatory response to pathogenic microorganisms, which can be fatal (Sepsis 3.0)^3^. In the last decade, the incidence rate of sepsis was 437 per 100,000 person-years, and the incidence of severe sepsis (as defined by Sepsis 2.0^2^) was 270 per 100,000 person-years in developed countries^4^. Fleischmann et al.^4^ also inferred that there were 31.5 million sepsis cases per year and 19.4 million severe sepsis cases per year worldwide, with 5.3 million deaths annually. The case-fatality rate can reach up to 30% in sepsis, 50% in severe sepsis and 80% in septic shock^5^. In addition, the prevalence of sepsis and the contribution of sepsis to all-cause mortality rates have been increasing in the last several years^6^. Severe sepsis in elderly patients was an independent risk factor for substantial and persistent new cognitive impairment and functional disability among survivors^7^. Delays in treatment and inappropriate antibiotic therapy dramatically reduce survival rates in septic shock^8,9^. Hence, the early diagnosis of sepsis is particularly important. At present, the diagnosis of sepsis is difficult and complicated. Although there are clinical guidelines and many laboratory tests to diagnose sepsis, e.g., the C-reactive protein (CRP) test, procalcitonin (PCT) test, and white blood cell (WBC) count, their specificity is unsatisfactory. Blood culture provides conclusive evidence for the diagnosis of sepsis, but the sensitivity of blood culture is very low, and the process is time consuming, usually delaying diagnosis. On the other hand, SIRS patients can be misdiagnosed with sepsis because the symptoms and signs of SIRS are very similar to those of sepsis when the blood culture is negative; the misdiagnosis of SIRS as sepsis leads to antibiotic misuse and possible selection for drug resistance. The major differences between sepsis and SIRS include organ failure, which is assessed mainly by the Sequential (sepsis-related) Organ Failure Assessment (SOFA) score, and infectious states, measured by biological phenotype and clinical symptoms^3^. Biomarkers are defined as measurable and quantifiable biological parameters, which can be molecules, genes, proteins or other variables. There are hundreds of biomarkers, but only a small fraction of these are useful biomarkers for sepsis^10^, e.g., CRP, PCT, serum amyloid A (SAA), and triggering receptors expressed on myeloid cells-1 (TREM-1). Biomarkers are a promising way to diagnose sepsis, and this approach can facilitate early and accurate diagnosis, forecast organ dysfunction and assist in defining appropriate therapeutic plans^11^. Human urine contains thousands of proteins^12,13^ and extracellular vesicles^14^, which could be good resources to use as biomarkers. The method of isobaric tags for relative and absolute quantification (iTRAQ) is a mass spectrometry (MS)-based relative proteomic quantification method utilizing the derivatization of primary amino groups in intact proteins and isobaric tags for different peptide fragments^15,16^. iTRAQ can be used to screen the differentially expressed proteins among eight samples simultaneously and is highly sensitive and specific^17^. In this study, we sought to identify new diagnostic biomarkers of sepsis in urine by utilizing iTRAQ and to verify the biomarkers using enzyme-linked immunosorbent assays (ELISAs). In addition, we explored the diagnostic value of the combined biomarkers through logistic regression to enhance the diagnostic sensitivity and specificity to sepsis and help clinicians determine the appropriate therapeutic strategy.

## Methods and materials

### Urine samples and data collection

This study was performed at the Second Affiliated Hospital of Chongqing Medical University. The procedures used in this study were in accordance with the 2008 Declaration of Helsinki and approved by the Ethics Committee of the Second Affiliated Hospital of Chongqing Medical University (Grant no. 201916). The urine samples and patient data came from the Adult Multidisciplinary Intensive Care Unit (ICU) and Infection Department. All patients or their bailors were informed about the study procedures, risk and privacy policy, and written consent was signed by each participant. Urine samples were collected from patients in the sepsis group, SIRS group and healthy control group. Consecutive patients with sepsis or SIRS were enrolled, and the healthy control group was enrolled by recruitment. The sepsis inclusion criteria^3^ were as follows: (1) age 18 years or older, (2) proven infection or suspected infection as adjudicated retrospectively by three physicians, and (3) a SOFA score of 2 or more points. The specific criteria for the SOFA score are listed in Table 1^3,18^, and the effectiveness of the SOFA has been assessed^19^. The SIRS inclusion criteria^1^ were as follows: (1) temperature > 38 °C or < 36 °C; (2) heart rate > 90/min; (3) respiratory rate > 20/min or PaCO_2_ < 32 mmHg; (4) white blood cell count > 12 × 10^9^/L or < 4 × 10^9^/L or > 10% immature bands in peripheral blood; (5) proven lack of infection, or no suspected infection as adjudicated retrospectively by three physicians; and (6) age 18 years or older. The patients who were diagnosed with SIRS must have presented with two or more of the first four criteria. Healthy control inclusion criteria were that the healthy volunteer did not suffer from infectious disease or take antibiotics. The exclusion criteria were as follows: (1) immune deficiency, (2) autoimmune diseases, (3) pregnancy; (4) use of any antibiotics before hospitalization; and (5) refusal to take part in the study or refusal to provide written and signed consent. Urine samples were collected as soon as the clinical diagnosis was made, and the interval time was more than 4 h from the patients’ last micturition. The urine was collected in a 50 mL centrifuge tube directly from the bladder, and no protease inhibitor was added to the samples. The collected samples were transported in ice water mixture. These samples were centrifuged at 2000×*g* for 10 min to separate the cellular or tissue debris. Finally, the samples were divided into five 2.0 mL Eppendorf tubes and stored at −80 °C until analysis^20^. The patient data were collected through the electronic medical system of the Second Affiliated Hospital of Chongqing Medical University.

**Table 1:** Sequential (sepsis-related) organ failure assessment score^3,18^.

Score Criteria	0	1	2	3	4
PaCO_2_/FiO_2_ (mmHg)	≥ 400	< 400	< 300	< 200	< 100
Platelets (× 10^9^/L)	≥ 150	< 150	< 100	< 50	< 20
Bilirubin (μmmol/L)	< 20	20–32	33–101	102–204	> 204
MAP (mmHg)	≥ 70	< 70	Dopamine < 5 or dobutamine (any dose)^a^	Dopamine 5.1–15 or epinephrine ≤ 0.1 or norepinephrine ≤ 0.1^a^	Dopamine > 15 or epinephrine > 0.1 or norepinephrine > 0.1^a^
Glasgow Coma Scale Score^21^	15	13–14	10–13	6–9	6
Creatinine (mmol/L)	< 110	110–170	171–299	300–400	440
Urine output (ml/day)	–	–	–	< 500	< 200

*PaO2* partial pressure of oxygen, *FIO2* fraction of inspired oxygen, *MAP* mean arterial pressure.

^a^Catecholamine doses are given as μg/(kg min) for at least 1 h.

### Materials and measurement procedure

#### Protein extraction from urine and iTRAQ procedure

We collected 10 samples from each of the groups to perform a preliminary analysis of the differentially expressed proteins among the three groups. The samples were pooled by group (sepsis, SIRS, and healthy control), and the proteins were precipitated using two volumes of cooled acetone (2 h)^13^. Finally, the three samples were centrifuged at 25,000×*g* at 4 °C for 15 min, and the supernatants were discarded. This procedure was repeated twice.

The precipitates were air-dried and dissolved at room temperature in an appropriate amount of lysis buffer using an ultrasonic homogenizer. Finally, the solutions were centrifuged at 25,000×*g* at 4 °C for 15 min, and the supernatants were collected. The Bradford method was used to measure the protein concentrations.

An iTRAQ 8-Plex Reagent Kit (Applied Biosystems, Foster City, CA, USA) was used to label the protein. The prepared protein was precipitated, redissolved, alkylated, cysteine blocked and digested following the iTRAQ Kit instructions and the study by Wisniewski^22^. The sepsis group was labeled with tag 113, the SIRS group was labeled with tag 119, and the healthy control group was labeled with tag 121. The labeled peptide solutions were pooled and freeze-dried under vacuum prior to further analysis.

The peptides were fractioned as previously described^23,24^. The prepared peptides were analyzed on the TripleTOF 5600 system coupled to an Eksigent NanoLC-2D system (AB Sciex, Framingham, MA, USA), and each of the samples was analyzed twice. The data were processed by ProteinPilot V2.0 (Applied Biosystems) and searched against the UniProt human proteome database (https://www.uniprot.org/). Proteins with an Unused Protscore > 1.3, a fold change > 2 and a P value < 0.05 were defined as differentially expressed.

#### ELISA measurement procedure

We employed the Human AACT-a1 (ab217779, Abcam, UK), Human C Reactive Protein ELISA Kit (ab9995, Abcam, UK), Human PEGC ELISA Kit (ab275552, Abcam, UK), Human Glutaredoxin-1 (abx151683, Abbexa, UK), Human Haptoglobin ELISA (ab219048, Abcam, UK), Human HLA-II histocompatibility antigen (abx387810, Abbexa, UK), Human LRG1 ELISA Kit (NBP2-60577, Novus Biologicals, Centennial, CO, USA), Human Resistin ELISA Kit (ab183364, Abcam, UK) and Human Serum Amyloid A ELISA Kit (KT-547, Kamiya Biomedical Company, Seattle, WA, USA) to measure the concentrations of target proteins. Urine samples from each patient were measured by ELISA, and the measurement protocol was performed according to the instructions of the three respective ELISA kits. The specifics of each ELISA kit are listed in Table 2. Duplicate standards or samples were set, and the mean absorbance of duplicate wells was calculated. The concentration of urinary proteins (CRP, LRG1, SAA) was normalized to urinary creatinine (u-Cr) and expressed as protein/u-Cr in μg/mmol to adjust for individual differences and potential kidney injury^25,26^. All statistical analyses were performed using Microsoft Excel 2019 (Microsoft Corporation, Redmond, WA, USA) and SPSS 25 (International Business Machines Corporation, Armonk, NY, USA).

**Table 2:** The characteristics of ELISA kit.

Trait Reagent	Sensitivity	Working range	Coefficient of variation (CV, %)	
			Intra-assay	Inter-assay
AACT-a1 ELISA Kit	327 pg/ml	1176-40,000 pg/ml	< 4	< 4.3
CRP ELISA Kit	2 pg/ml	34.29–25,000 pg/ml	< 10	< 12
PGC ELISA Kit	16.5 pg/ml	54.69–3500 pg/ml	< 2.8	< 8.1
Glutaredoxin-1 ELISA Kit	28.9 pg/ml	62.5–4000 pg/ml	< 4.8	< 6.2
Haptoglobin ELISA Kit	86 pg/ml	312.5–20,000 pg/ml	< 1.8	< 4.4
HLA-II histocompatibility antigen ELISA Kit	0.188 ng/ml	0.313–20 ng/ml	< 5	< 6
LRG1 ELISA Kit	0.17 ng/ml	0.313–20 ng/ml	< 3.1	< 9.4
Resistin ELISA Kit	24 pg/ml	78.1–5000 ng/ml	< 3	< 6
SAA ELISA Kit	0.50 ng/ml	1.56–100 ng/ml	< 8.5	< 11

## Results

### Subject characteristics

There were a total of 151 subjects included in our study: 53 sepsis patients, 48 SIRS patients and 50 healthy volunteers. The average ages of the three groups were 58.4, 62.0 and 51.0 years. The percentages of males were 64.2%, 75%, and 56%, respectively. In the sepsis group, there were 24 patients whose blood cultures were positive. The detected bacteria consisted of *Escherichia coli* (10, 41.7%), *Staphylococcus aureus* (6, 25%), *Enterococcus faecium* (3, 12.5%), *Acinetobacter baumannii* (2, 8.3%), *Streptococcus* (1, 4.2%), *Lactobacillus brevis* (1, 4.2%) and *Klebsiella pneumoniae* (1, 4.2%). There were no statistically significant differences in the distributions of age or gender among the three groups. The distribution of WBC count and neutrophil percentage were statistically similar and could not distinguish the sepsis and SIRS patients in this study. In addition, the sepsis patients presented higher urinary creatinine levels and pulse rates than the healthy control group. Patient data are summarized in Table 3.

**Table 3:** Subject characteristics and quantitative analysis.

	Sepsis (n = 53)	SIRS (n = 48)	Healthy (n = 50)	P value
Age	62.0 (47.0, 73.0)	65.0 (49.8, 71.3)	61.0 (48.5, 70.5)	NS^a, b, c^
Males, n (%)	34 (64.2)	36 (75)	28 (56)	NS*
WBC (× 10^9^/L)	10.3 (6.9, 13.1)	11.5 (8.0, 14.0)	N	NS^a^
Neutrophil (%)	86.7 (82.5, 90.2)	86.0 (81.0, 89.0)	N	NS^a^
SOFA score	8 (2, 17)	0.7 (0, 1)	0	P < 0.001^a^
s-CRP (mg/L)	149.5 (105.1, 183.9)	59.7 (21.1, 80.6)	N	P < 0.001^a^
s-Cr (μmol/L)	78.5 (53.9, 137.2)	55.7 (45.0, 71.6)	N	P < 0.001^a^
u-Cr (mmol/L)	4.9 (3.5, 7.8)	4.4 (3.6, 5.5)	4.4 (3.3, 5.3)	NS^a, c^, P = 0.041^b^
T (℃)	38.0 (37.2, 38.8)	38.0 (36.4, 41.0)	N	NS^a^
R (/min)	20 (16, 22)	21 (14, 32)	N	NS^a^
P (/min)	110 (90, 124)	100 (74, 140)	N	P = 0.03^a^
uCRP (μg/L)	6738.2 (4043.5, 10,393.9)	2050.8 (637.6, 3283.5)	10.6 (8.0, 13.1)	P < 0.001^a, b, c^
uLRG1 (μg/L)	7701.8 (4835.6, 16,180.7)	2724.6 (1945.3, 4676.8)	55.5 (52.5, 61.5)	P < 0.001^a, b, c^
uSAA (μg/L)	29.0 (23.8, 44.9)	14.3 (8.8, 18.5)	0.32 (0.30, 0.38)	P < 0.001^a, b, c^
uCRP/u-Cr (μg/mmol)	1337.6 (773.8, 1822.3)	358.7 (149.8, 786.5)	2.4 (1.8, 3.3)	P < 0.001^a, b, c^
uLRG1/u-Cr (μg/mmol)	1614.4 (1197.3, 2145.1)	644.5 (469.5, 1077.7)	13.6 (10.7, 18.6)	P < 0.001^a, b, c^
uSAA/u-Cr (μg/mmol)	6.3 (4.7, 8.8)	2.9 (1.7, 4.1)	0.079 (0.061, 0.108)	P < 0.001^a, b, c^

Variables are expressed as median (25% percentiles, 75% percentiles); Mann–Whitney U test or chi-square test were performed to test the statistical significance between different groups; a, tested by Mann–Whitney U test between the sepsis group and SIRS group; b, tested by Mann–Whitney U test between the sepsis group and healthy control group; c, tested by Mann–Whitney U test between SIRS group and healthy control group; *, tested by chi-square test; Significance level is P < 0.05.

*WBC* white blood cell, *s-CRP* serum C-reactive protein, *s-Cr* serum creatinine, *u-Cr* urinary creatine, *uCRP* urinary CRP, *uLRG1* urinary LRG1, *uSAA* urinary SAA, *T* body temperature, *R* respiratory rate, *P* pulse rate, *N* none data.

### iTRAQ identification of differentially expressed proteins

**Figure 1:**
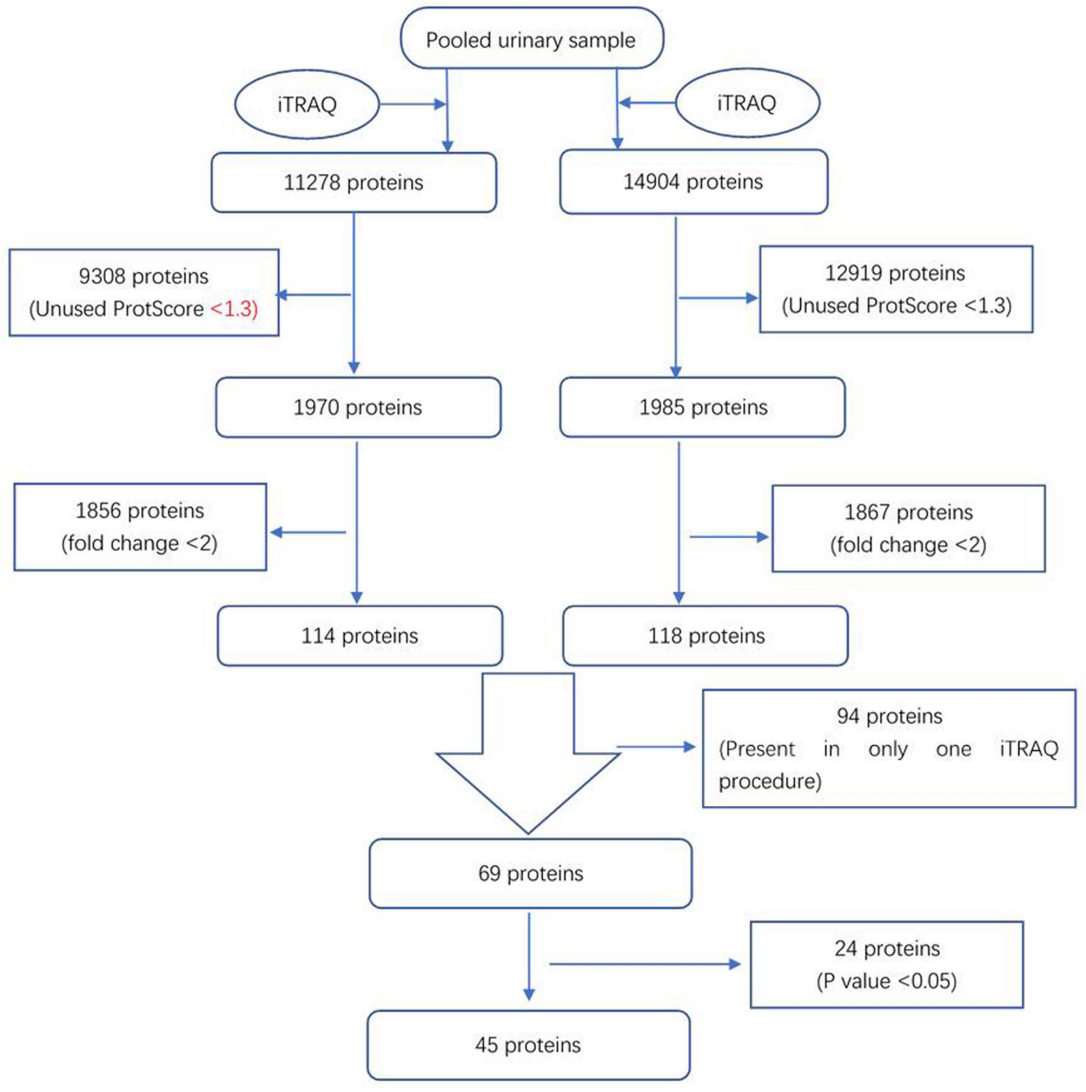
Flow chart of protein identification.

**Figure 2:**
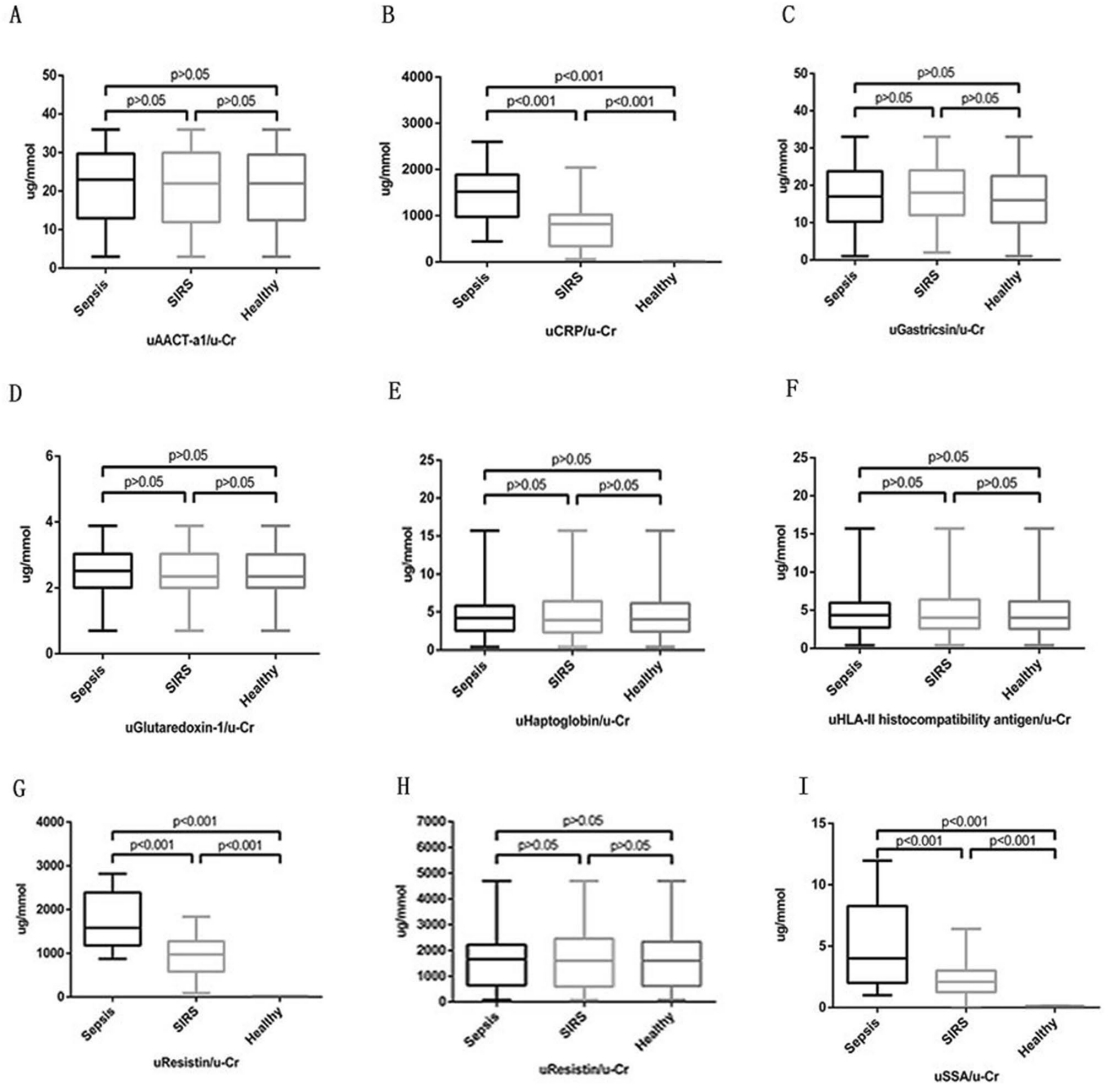
Box plot of adjusted concentrations.

iTRAQ identified 11278 and 14904 proteins in duplicate analyses. There were 1970 and 1985 proteins with Unused ProtScore values > 1.3. Among these proteins, there were 114 and 118 with fold change > 2 between the sepsis group and healthy control group. There were 69 proteins that were detected in both iTRAQ procedures. After eliminating the proteins with P values > 0.05, 45 proteins met the screening criteria. The flow chart is presented in Fig. 1. Nine differentially expressed proteins that might be related to sepsis and SIRS were tested further by ELISA, and CRP, LRG1, and SAA were chosen as the target biomarkers (Fig. 2). Their iTRAQ information are listed in Table 4. The blood levels of these three proteins have previously been found to be related to sepsis and SIRS^27–29^.

**Table 4:** iTRAQ information of identified biomarkers.

	CRP	LRG1	SAA
Accession	sp|P02741|CRP_HUMAN	sp|P02750|A2GL_HUMAN	tr|D3DQX7|D3DQX7_HUMAN
Unused	115.64	59.08	83.08
113:119	2.68	3.49	2.75
P value (113:119)	1.81E−12	0.000162	6.47E−5
119:121	6.93	4.14	7.26
P value (119:121)	3.81E−11	2.96E−7	5.88E−9

### Quantitative and statistical analysis

The concentrations of uCRP, uLRG1 and uSAA are shown in Table 3. Considering the variability of glomerular filtration rate (GFR), 24-h urinary volume and glomerular filtration barrier among the different subjects, the concentrations normalized to urinary creatinine levels were also listed as uCRP/u-Cr, uLRG1/u-Cr and uSAA/u-Cr in μg/mmol and were used for further statistical analyses. The normality of acquired data was tested, and we found that not all the data followed a Gaussian distribution. Although efforts were made to transform the data to a Gaussian distribution by converting initial data to the square root or logarithm, the data did not conform. Therefore, the data are presented as the median (25th percentile, 75th percentile), and nonparametric tests were used to analyze the statistical characteristics. Spearman's rank correlation coefficient was calculated (Table 5). Serum CRP was strongly correlated with urine CRP, which may indicate that urinary CRP came from the blood. In addition, the three biomarkers (uCRP, uLRG1, uSAA) were pairwise correlated, which may indicate that they had similar status during the infection process. The median values were highest in the sepsis patients, intermediate in the SIRS patients and lowest in the healthy control group (P < 0.001).

**Table 5:** Spearman's rank correlation coefficient.

	uCRP	uLRG1	uSAA	sCRP
uCRP	1			
uLRG1	0.822^a^	1		
uSAA	0.853^a^	0.829^a^	1	
sCRP	0.846^a^	0.493^a^	0.493^a^	1

^a^The P value is < 0.001.

**Figure 3:**
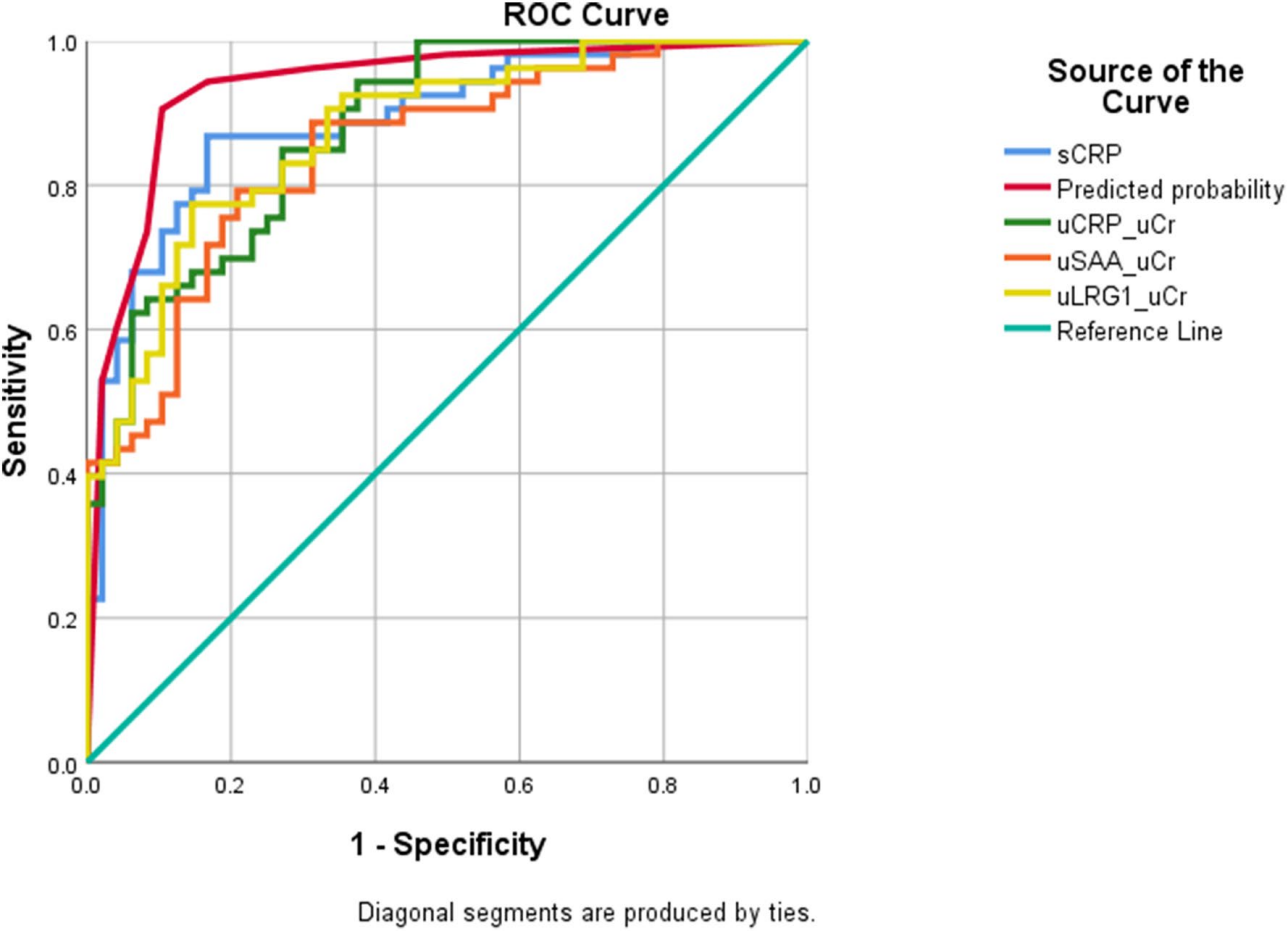
The ROC curves of adjusted concentration, s-CRP and predicted probability.

When these biomarkers were adjusted to urine creatinine, the same conclusion could be drawn; their values are presented in Fig. 2. For the adjusted concentrations and s-CRP, receiver operating characteristic curves (ROC) were drawn by SPSS 25 (Fig. 3). The area under the curve values were 0.878 (uCRP/u-Cr) and 0.874 (uLRG1/u-Cr) and 0.849 (uSAA/u-Cr) and 0.891 (s-CRP). The Youden index (sensitivity + specificity – 1) was used to determine the best cutoff value. The sensitivity, specificity, cutoff value and area under the curve values are listed in Table 6. s-CRP had the highest diagnostic efficiency. The diagnostic difference was small among uCRP/u-Cr, uLRG1/u-Cr and uSAA/u-Cr. The adjusted concentrations (uCRP/u-Cr, uLRG1/u-Cr and uSAA/u-Cr) of the sepsis and SIRS groups were transformed to binary variables based on whether they were greater than the cutoff value and further analyzed by logistic regression. The forward stepwise (likelihood ratio) method was employed to process the data. The omnibus test was used to verify the statistical significance (P < 0.001), and the Hosmer and Lemeshow test showed that the model had taken full advantage of the acquired data (P = 0.712). The regression coefficient and P values are listed in Table 7. According to the regression results, all three biomarkers were included in the regression model. If the patients’ parameters exceeded the cutoff value, the corresponding risk of sepsis would increase 9.913-fold (uCRP/u-Cr), 15.936-fold (uLRG1/u-Cr) and 12.793-fold (uSAA/u-Cr) compared to SIRS patients. The sepsis risk score was logit(P) = 2.294*X_1_ + 2.734*X_2_ + 2.549*X_3_-3.714 (X_1_ = 1, if uCRP/u-Cr > 746.1 μg/mmol; X_1_ = 0, if uCRP/u-Cr > 746.1 μg/mmol; X_2_ = 1, if uLRG1/u-Cr > 1174.8 μg/mmol; X_2_ = 0, if uLRG1/u-Cr < 1174.8 μg/mmol; X_3_ = 1, if uSAA/u-Cr > 4.4 μg/mmol; X_3_ = 0, if uSAA/u-Cr < 4.4 μg/mmol). According to logit(P), the prediction probabilities were calculated by P = e^logit(P)^/(1 + e^logit(P)^), and its ROC curve was also drawn (Fig. 3). The area under the curve was 0.937. The cutoff of logit(P) was 0.514. Its sensitivity and specificity were 0.906 and 0.896, respectively. This indicated that the sensitivity and specificity of diagnosis would be greatly enhanced by combining the panel of biomarkers.

**Table 6:** The diagnostic value of identified biomarkers.

	Area	Cut-off (μg/mmol)	Sensitivity	Specificity	Youden index
uCRP/u-Cr	0.878	746.1	0.849	0.729	0.578
uLRG1/u-Cr	0.874	1174.8	0.774	0.854	0.628
uSAA/u-Cr	0.849	4.4	0.792	0.792	0.584
sCRP	0.891	87.76	0.868	0.833	0.701

**Table 7:** Variables in the equation.

	B	Sig	Exp(B)	95% C.I. for Exp(B)	
				Lower	Upper
uCRP/u-Cr	2.294	0.001	9.913	2.608	37.677
uLRG1/u-Cr	2.734	< 0.001	15.396	3.307	49.489
uSAA/u-Cr	2.549	< 0.001	12.793	3.926	60.380
Constant	-3.714	< 0.001	0.024	–	–

*B* regression coefficient, *Sig.* statistical significance, *exp(B)* equal to odds ratios, *95% C.I.* refers to 95% confidence interval.

## Discussion

Because the symptoms of sepsis and SIRS are quite similar, it is frequently difficult to distinguish a septic infection from SIRS. After Sepsis 3.0 was issued, the situation became more difficult, in part because patients suffering from infection-related SIRS, but without organ dysfunction, can be confused with patients who have SIRS without sepsis. Biomarkers, especially acute-phase proteins, could be key to distinguishing sepsis from SIRS, but their specificity has been unsatisfactory, making it necessary to combine multiple biomarkers to enhance overall specificity^28,29^. The three biomarkers identified in the present study were all acute-phase reaction proteins^30–35^. CRP was identified in the 1930s and is synthesized in the liver and released in response to infection, trauma and immune disorders. The mean s-CRP levels were 98 mg/L in sepsis patients and 70 mg/L in SIRS patients^36^. Most recently, bacterial infection is suspected when s-CRP is greater than 100 mg/L in the clinical context. Conversely, the absence of severe bacterial infection is indicated when s-CRP is less than 20 mg/L^33^. In the present study, we first verified the existence of CRP in urine, and uCRP/u-Cr was strongly related to s-CRP. Although the diagnostic efficiency of uCRP/u-Cr was slightly lower than that of s-CRP, uCRP/u-Cr could still be a good substitute for s-CRP, considering the convenience and noninvasiveness of specimen collection and the strong relationship between the urinary and serum concentrations. Leucine-rich α-2 glycoprotein 1 (LRG1), first identified in 1977^37^, is synthesized by hepatocytes, stored in neutrophils^38^ or myeloid cells^39^, and released into the serum when the body is in the acute phase of stimulation by bacterial or viral infection^40^. LRG1 belongs to the leucine-rich repeat (LRR) protein family and plays a role in protein interactions, innate immunity, platelet aggregation and angiogenesis. LRG1 is usually used as a biomarker of tumors, appendicitis, diabetes complications, and inflammatory disease^41–44^. According to our measurement results, uLRG1/u-Cr of sepsis patients was obviously higher in sepsis patients than in either SIRS patients or healthy volunteers (P < 0.001), indicating that urinary LRG1 is a promising biomarker in diagnosing sepsis and distinguishing sepsis from SIRS. Considering the lower sensitivity (0.774) and higher specificity (0.854), LRG1 would be a good choice to combine with other biomarkers. Serum amyloid A protein is also a well-known acute-phase protein produced by the liver. Its concentration is very low (1 μg/mL) in healthy people but can increase dramatically (100-fold or more) when the human body is in the acute phase^45^. Human SAA consists of 4 isotypes (SAA1, SAA2, SAA3, SAA4)^46^, but the sequence identity of SAA1 and SAA2 is more than 93%, and SAA1 is the main isotype in serum^47^. The SAA ELISA kits we employed in the current study were not sensitive to the SAA isotypes, and the concentration represented the concentration of total SAA. SAA is regulated by cytokines (interleukin-1β, interleukin-6, tumor necrosis factor, etc.) and regulate cytokines (interleukin-23, interleukin-33, interleukin-10, etc.) which is summarized as the cytokine-SAA-chemokine network^48^. Considering its interaction with cytokines, it is believed that SAA takes part in inflammatory diseases, angiogenesis and tumor growth, as do other acute-phase proteins^49–51^. In our study, the sensitivity and specificity of uSAA/u-Cr were between those of uCRP/u-Cr and uLRG1/u-Cr. This was also the first time the value of uSAA for sepsis diagnosis and differential diagnosis was verified.

In the current study, a range of inflammatory biomarkers were measured in patients with sepsis and SIRS. CRP is a nonspecific indicator of systemic inflammation, and patients with noninfectious causes of SIRS may also have markedly increased serum concentrations of this biomarker. The sensitivity and specificity of CRP for differential diagnosis between sepsis and SIRS were lower than those of the three combined biomarkers in our study^52^. PCT has been used to differentiate sepsis from SIRS^53^, but patients with systemic inflammation of noninfectious etiology may have increased levels of PCT in some cases^54^. Therefore, the diagnostic utility of measurement of CRP and PCT should be considered combined with other clinical and laboratory information in the clinical setting. In addition to CRP and PCT, the concentrations of the circulating anti-inflammatory cytokines IL-Ra and IL-10 were found to be significant in discriminating between sepsis and SIRS^55–57^. The diagnostic accuracy of urinary orosomucoid (u-ORM) for sepsis (AUC ROC: 0.954) was similar to that of PCT^58^. Few studies have been performed regarding urinary GSN (u-GSN) levels in sepsis. Maddens et al. reported increased u-GSN levels in septic mice but did not predict the diagnostic value of u-GSN in sepsis^59^. The diagnostic value of these biomarkers was lower than that of the three parameters in our study. However, in different studies, different experimental methods and populations have an impact on the experimental results. Therefore, larger samples and more in-depth studies are needed to compare the diagnostic utility of these indicators.

Although the three biomarkers were normalized to urinary creatinine in the statistical process, adjusting the concentration had only a small influence on the statistical results and no influence on the final conclusion. When the panel of biomarkers was combined, the sensitivity to sepsis was increased to 0.906, and the specificity was increased to 0.896, which greatly exceeded any single biomarker. The area under the ROC curve was 0.937, which indicated favorable diagnostic efficiency. Serum CRP data were acquired through the medical electronic system, but serum LRG1 and SAA were not available by routine examination at the Second Affiliated Hospital of Chongqing Medical University. Hence, serum LRG1 and SAA data were lacking in our study. The correlation between serum LRG1 and urinary LRG1 was not verified, nor was the correlation of serum SAA and urinary LRG1. According to the correlation between serum CRP and urinary CRP, we inferred that urine CRP came from serum CRP. Considering the similar mechanism of protein excretion by the kidney, we hypothesized that urinary LRG1 and SAA also came from the blood. There were only 101 patients included in our study, and it would be necessary to recruit more patients to further verify the efficiency of the combined biomarkers. The logistic regression model represented only a “discovery” finding, and we did not use K-fold cross-validation or other forms of cross-validation; thus, unfortunately, we must regard it as preliminary. In addition, the diagnostic value of LRG1 for sepsis was not fully investigated, and additional research is necessary to further understand the diagnostic significance of LRG1.

## Conclusions

C-reactive protein, serum amyloid A protein and leucine-rich alpha glycoprotein-1 are present in urine. Urinary C-reactive protein comes from blood and can substitute for serum C-reactive protein in sepsis diagnosis. Urine SAA and leucine-rich alpha glycoprotein-1 were also useful biomarkers for sepsis diagnosis. This initial study suggests the possibility of improved differential diagnosis between sepsis and systemic inflammatory response syndrome, and further confirmation is necessary to further confirm the findings (Suppl. information).

## Supplementary Information

Supplementary Information.

## Abbreviations

SIRS: Systemic inflammatory response syndrome
CRP: C-reactive protein
uCRP: Urinary CRP
PCT: Procalcitonin
SAA: Serum amyloid A protein
uSAA: Urinary SAA
TREM-1: Triggering receptors expressed on myeloid cells-1
iTRAQ: Isobaric tags for relative and absolute quantification
ELISA: Enzyme linked immunosorbent assay
ICU: Intensive care unit
SOFA: Sequential (sepsis-related) organ failure assessment
LRG1: Leucine-rich alpha glycoprotein-1
uLRG1: Urinary LRG1
u-Cr: Urinary creatinine
CV: Coefficient of variation
SPSS: Statistical product and service solutions
GFR: Glomerular filtration rate
ROC: Receiver operating characteristic curve
LRR: Leucine rich repeat
